# Life strategy and grazing intensity responses of *Brachionus calyciflorus* fed on different concentrations of microcystin-producing and microcystin-free *Microcystis aeruginosa*

**DOI:** 10.1038/srep43127

**Published:** 2017-02-23

**Authors:** Ye Liang, Kai Ouyang, Xinglan Chen, Yuqi Su, Jiaxin Yang

**Affiliations:** 1 Jiangsu Province Key Laboratory for Biodiversity & Biotechnology and Jiangsu Province Key Laboratory for Fisheries Live Food, School of Life Sciences, Nanjing Normal University, Nanjing, 210023, P. R. China

## Abstract

The occurrence of *Microcystis* blooms is a worldwide concern due to the numerous adverse effects on zooplankton. We therefore hypothesized that the cyanobacterium *Microcystis aeruginosa* is harmful to rotifer growth. Population and individual experiments were conducted with the same proportional volumes of *Chlorella* and *Microcystis* for given food densities. Life-table parameters, life-history traits, and the grazing intensity of *Brachionus calyciflorus* were evaluated after they had fed on microcystin-producing and microcystin-free *Microcystis*, both alone and combined with an edible alga (*Chlorella pyrenoidosa*), at concentrations of 1 × 10^5^, 1 × 10^6^, and 1 × 10^7^ cells mL^−1^. The results showed that the interactive effects of food density and type appeared to be synergistic on generation time (T), net reproduction rate (R_0_), body length, swimming speed, and reproduction time. In contrast, these effects appeared to be antagonistic on intrinsic growth rate (r), finite rate of increase (λ), time to first brood, post-reproductive time and total offspring per female. The grazing rate of rotifers decreased with grazing time. Although the toxins released after grazing on *M. aeruginosa* had negative effects on rotifer growth and reproduction, *B. calyciflorus* changed its life strategy and grazing intensity in response to eutrophic conditions.

Cyanobacterial blooms are a growing problem worldwide, resulting in increasing concentrations of the cyanobacterium *Microcystis* in eutrophic lakes and reservoirs, significantly impacting entire aquatic ecosystems^1^. The concentration of *Microcystis* can be as high as 10^7^ cells mL^−1^ in eutrophic water bodies when there is a serious cyanobacterial bloom outbreak^2^. This has been observed in Lake Taihu, which is typical of many lakes in the Asian subcontinent, and was especially apparent during the Lake Taihu water crisis in 2007^3^. There are three forms of cyanobacteria (unicellular, filamentous, and colonial) that affect zooplankton growth and reproduction^4^,^5^. Given their edible size range, unicellular species are more toxic to aquatic animals than are large cyanobacterial species^4^. Laboratory investigations revealed that filaments and colonies of cyanobacteria mechanically interfere with the grazing rate of grazers by reducing the ingestion of food particles^6^. *Microcystis aeruginosa* reduces the survival and reproduction of zooplankton in various ways (e.g., poor nutritional value, toxicity, and morphology); thus, various studies have investigated the interactions of cyanobacteria with zooplankton, and their effect on ecosystems^7^,^8^,^9^,^10^.

Rotifers are a dominant life form in freshwater bodies and are the natural food link between primary producers (algae) and zooplanktivorous fish. They also have an important role in maintaining the ecological balance in freshwater ecosystems^11^. *Brachionus calyciflorus* is usually used as a model organism for ecotoxicological investigations^12^,^13^. Following a breakout of *M. aeruginosa*, rotifers change their life strategies (e.g., growth, reproduction, body length, swimming speed, etc.) in response to the harsher aquatic environment. The effects of toxic cyanobacteria that produce secondary metabolites that suppress the population growth and reproduction of rotifers have been well demonstrated^14^. An overall collapse of *Brachionus* populations was observed within 3 days of being fed toxic *M. aeruginosa*^15^. Studies of factors affecting rotifer population growth have mainly focused not only on the algae type and species, but also on particle density^16^,^17^. The population growth rate of rotifers was reported to be reduced by half with increasing concentrations of *Microcystis*^15^. There are few published experimental studies of the effects of food concentration and food type on the ecological toxicology parameters of rotifers. The studies discussed above used a microcystin-producing strain of *Microcystis* as the sole bioassay organism. However, microcystin-free *M. aeruginosa* are also likely to influence zooplankton, given that toxic and nontoxic strains of *M. aeruginosa* coexist in natural aquatic ecosystems^18^. Generally, *M. aeruginosa* do not release extracellular toxins; therefore, only those zooplankton species that ingest *Microcystis* should be affected by the toxins released as the cells lyse. The susceptibility of rotifers to *M. aeruginosa* is partly because of their tendency to feed on *Microcystis*^14^.

The study aimed to determine how microcystin-producing and micorcystin-free *Microcystis* affected the life history characteristics and grazing rates of *B. calyciflorus* compared to *Chlorella*. We hypothesized that food type and food density have different effects on rotifer life strategy, and toxic *Microcystis* was predicted to have negative effects on its life history characteristics. There are a priori reasons to expect the two factors to affect some life history parameters. Alva-Martínez *et al*. reported that population growth rates were inversely related to an increasing proportion of *M. aeruginosa* in the diet^19^. Three different life history characteristics of females in *B. calyciflorus* (e.g., time to first brood, reproductive time, and post-reproductive time) were found to have different responses to algae types^20^.

This study investigated whether the toxic effects of *M. aeruginosa* on *B. calyciflorus* were the result of the action of cyanobacterial cells after they had been ingested by the rotifers. The study focused mainly on evaluating the life-strategy and grazing-intensity responses of *B. calyciflorus* fed microcystin-producing and microcystin-free *M. aeruginosa*, both alone and combined with the green alga *Chlorella pyrenoidosa*. Population and individual experiments were conducted to assess the rotifer life strategy. The grazing rate of *B. calyciflorus* was investigated to determine any cyanobacteria–rotifer interactions. Comparing the growth, reproduction, and grazing intensity of rotifers fed on different food concentrations and types will improve our understanding of the impact of toxic materials on cyanobacteria–rotifer interactions and the mechanisms whereby cyanobacteria affect the zooplankton community.

## Results

### Changes in rotifer life-table parameters

The survivorship data of rotifers in the life-table parameter experiment are shown in Table 1. The incipient rotifer number was 10 in the population-based experiment. *Brachionus calyciflorus* lived longer in the *Chlorella* group than in other treatments at all food concentrations. The rotifer population had a maximum survival time of 144 h, which occurred in the C_p_ group at the concentration of 1 × 10^6^ cells mL^−1^. Food type and density had negative effects on rotifer survival. The rotifer population lasted 72 h in the mixture of toxic *M. aeruginosa* and *C. pyrenoidosa*, while it lasted 96 h in single *Microcystis* at the concentration of 1 × 10^6^ cells mL^−1^. The population lasted 72 h in toxic *Microcystis* treatments, while it lasted 96 h in nontoxic *Microcystis* groups at the concentrations of 1 × 10^5^ and 1 × 10^7^ cells mL^−1^.

**Figure 1:**
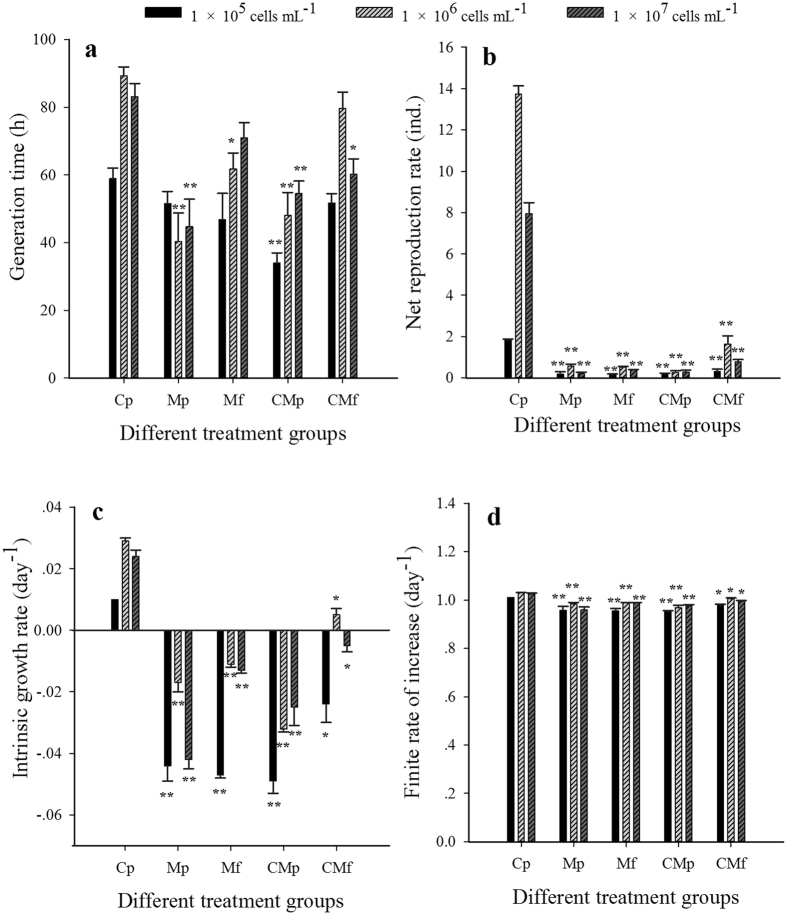
Changes in rotifer life-table parameters under different food concentrations and types. The life-table parameters are (**a**) generation time (T), (**b**) net reproduction rate (R_0_), (**c**) intrinsic growth rate (r), and (**d**) finite rate of increase (λ). Error bars indicate 1 SE (some error bars are too small to be visible). The significance designations mean that there were differences between food types for a given food density (*P < 0.01, **P < 0.05). All treatments were compared with *Chlorella*.

As determined by two-way analysis of variance (ANOVA), different concentrations and compositions of *M. aeruginosa, C. pyrenoidosa*, and the combination thereof, decreased the rotifer generation time (T) (*F* = 3.3, df = 8, *P* < 0.01) and net reproduction rate (R_0_) (*F* = 21.8, df = 8, *P* < 0.01). The interactive effects of food concentration and food type appeared to be synergistic on T and R_0_ (*P* < 0.01). Single dietary factors had negative effects on intrinsic growth rate (r) and finite rate of increase (λ) (*P* < 0.01), but the interactive effects of food concentration and food type did not result in any statistically significant differences in r (*F* = 1.1, df = 8, *P* > 0.05) or λ (*F* = 1.0, df = 8, *P* > 0.05). The r of rotifers fed *M. aeruginosa* mixtures at 1 × 10^5^ and 1 × 10^7^ cells mL^−1^ was negative, and decreased by more than 200% at a *M. aeruginosa* concentration of 1 × 10^5^ cells mL^−1^ compared with the C_p_ group (Fig. 1c). The minimum values of T (34.0 ± 5.1 h), R_0_ (0.2 ± 0.1 ind.), r (−0.2 ± 0.1 day^−1^), and λ (0.9 ± 0.1 day^−1^) occurred at a *M. aeruginosa* concentration of 1 × 10^5^ cells mL^−1^ (Fig. 1). Few rotifers survived and even fewer neonates were produced by the animals fed *M. aeruginosa* alone. *B. calyciflorus* fed microcystin-free *M. aeruginosa* lived longer than those fed on the microcystin-producing strain (Fig. 1a). In contrast, rotifers grew well and had high survival rates in the 100% *C. pyrenoidosa* treatment group (C_p_-group), and produced many neonates (Fig. 1b).

The one-way ANOVA revealed a significant food-type effect at each food concentration (*P* < 0.05). Toxic and nontoxic cyanobacteria inhibited the changes of rotifer life-table parameters. The T of *B. calyciflorus* cultured in 100% microcystin-free *M. aeruginosa* (M_f_-group) decreased at the *M. aeruginosa* concentration of 1 × 10^6^ cells mL^−1^ (*F* = 10.2, df = 14, *P* < 0.05). The same downward trend occurred in the 50% *C. pyrenoidosa* +50% microcystin-free *M. aeruginosa* group (CM_f_-group) at the concentration of 1 × 10^7^ cells mL^−1^ (*F* = 8.2, df = 14, *P* < 0.05). T also significantly decreased in the 50% *C. pyrenoidosa* +50% microcystin-producing *M. aeruginosa* group (CM_p_-group) at each food concentration and in the 100% microcystin-producing solution group (M_p_-group) at the concentrations of 1 × 10^6^ and 1 × 10^7^ cells mL^−1^ (*P* < 0.01, Fig. 1a). The reduction was as much as 11–55% compared with the C_p_ group. R_0_, r, and λ all declined in each *M. aeruginosa* group at the concentrations of 1 × 10^5^, 1 × 10^6^, and 1 × 10^7^ cells mL^−1^ (*P* < 0.05, Fig. 1b–d). Life-table parameter values declined by 2–590% for the different diets, indicating that both food concentration and food type had inhibitory effects on the growth of *B. calyciforus*.

### Rotifer life-history trait changes

**Figure 2:**
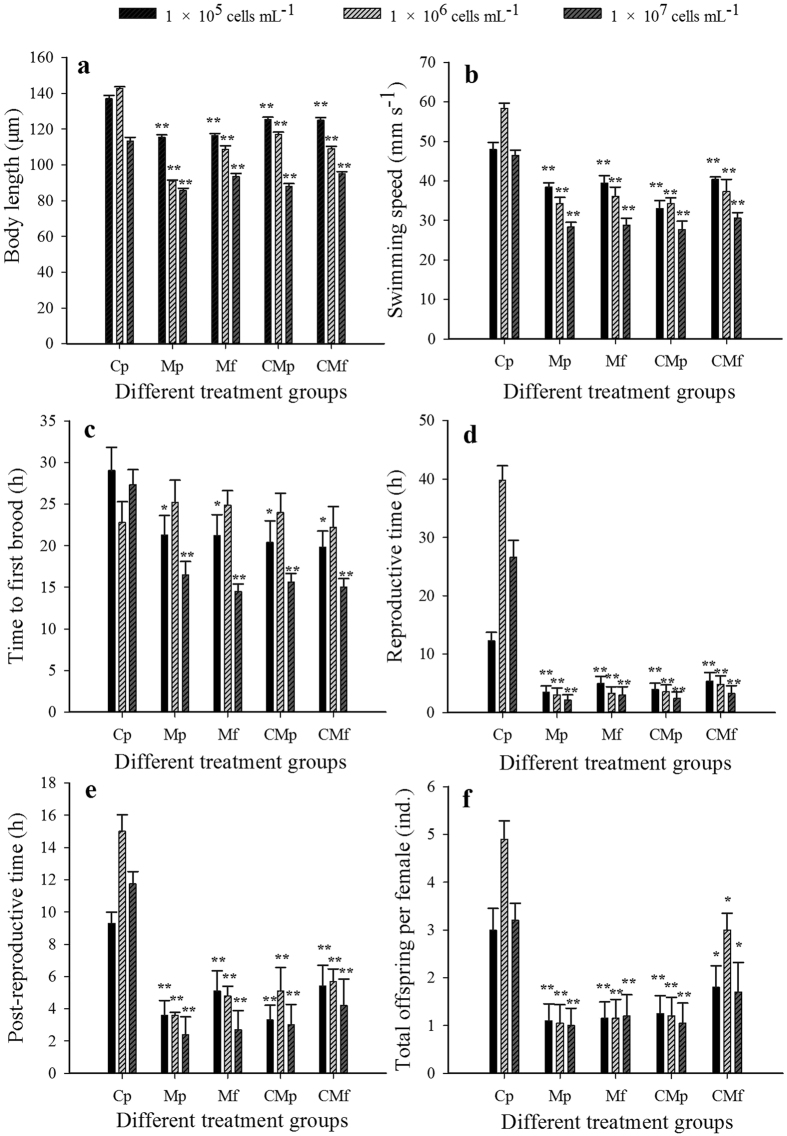
Changes in rotifer life-history traits under different food concentrations and types. The life-history traits are (**a**) body length, (**b**) swimming speed, (**c**) time to first brood, (**d**) reproductive time, (**e**) post-reproductive time, and (**f**) total offspring per female. Error bars indicate 1 SE. The significance designations mean that there were differences between food types for a given food density (*P < 0.01, **P < 0.05). All treatments were compared with *Chlorella*.

Variance analysis showed significant effects of food concentration and food type on rotifer life-history traits (two-way ANOVA, *P* < 0.01). The interactive effects of the two factors were synergistic on body length (*F* = 21.5, df = 8, *P* < 0.01), swimming speed (*F* = 3.1, df = 8, *P* < 0.01), and reproduction time (*F* = 17.8, df = 8, *P* < 0.01). The body length and swimming speed of *B. calyciflorus* decreased with the increasing concentration of *M. aeruginosa* (Fig. 2a,b). The rotifer time to first brood was affected by food concentration (*F* = 7.6, df = 2, *P* < 0.01) or food type (*F* = 4.0, df = 4, *P* < 0.01). Different strains of *M. aeruginosa* shortened the post-reproductive time (*F* = 20.5, df = 4, *P* < 0.01) and decreased the total number of offspring per female (*F* = 18.2, df = 4, *P* < 0.01).

A one-way ANOVA showed that the time to first brood of *B. calyciflorus* decreased in different cyanobacteria groups at the concentrations of 1 × 10^5^ cells mL^−1^ (*P* < 0.05) and 1 × 10^7^ cells mL^−1^ (*P* < 0.01) (Fig. 2c). The shortest time to first brood (14.5 ± 4.1 h) occurred in the M_f_-group at the concentration of 1 × 10^7^ cells mL^−1^. The toxic and nontoxic cyanobacteria significantly inhibited rotifer body length, swimming speed, reproductive time, and post-reproductive time (*P* < 0.05, Fig. 2a,b,d,e). These parameter values declined with increasing food concentration. The size of the reduction was as much as 16–92% at the concentration of 1 × 10^7^ cells mL^−1^. *B. calyciflorus* was more sensitive to toxic cyanobacteria. The minimum values of body length (85.7 ± 3.1 μm), swimming speed (33.3 ± 3.8 mm s^−1^), reproductive time (2.1 ± 4.0 h), and post-reproductive time (2.4 ± 4.9 h) occurred in the M_p_-group at the concentration of 1 × 10^7^ cells mL^−1^. These values were lower than the values in the C_p_-group (*P* < 0.01). Total offspring per female in the CM_f_-group was more (31–65%) than in the other groups. Greater reductions occurred in the M_p_, M_f_, and CM_p_ groups (58–79%, *P* < 0.01). The minimum value of total offspring per female (1.0 ± 0.9 ind.) occurred in the M_p_-group at a concentration of 1 × 10^7^ cells mL^−1^ (*P* < 0.01, Fig. 2f).

### Grazing intensity

The grazing rate of rotifers (G) varied significantly in the different *M. aeruginosa* groups within 60 min (two-way ANOVA, *P* < 0.01). In the single-diet groups, G increased with increasing food concentration. The minimum value of G (10 ± 1 cells ind^−1^ min^−1^) occurred in the M_f_-group at the concentration of 1 × 10^5^ cells mL^−1^ (Table 2). There was a high ingestion rate for *C. pyrenoidosa* in single and mixed diets (Tables 2 and 3). The G value of rotifers fed *C. pyrenoidosa* exceeded 7–76% compared to those fed unicellular cyanobacteria. In mixed solutions, the maximum G value for *Chlorella* was 4,472 ± 4 cells ind.^−1^ min^−1^, which occurred in the CM_p_ group at the concentration of 1 × 10^7^ cells mL^−1^ and at a grazing time of 15 min. The diameters of unicellular *C. pyrenoidosa* and *M. aeruginosa*, which are spherical in shape, are in the size range of particles that are suitable for digestion by *B. calyciflorus* (2–18 μm). The volume of *C. pyrenoidosa* (diameter: 3.7 ± 0.2 μm; volume: 27.4 ± 4.6 μm^3^) is smaller than that of microcystin-producing *M. aeruginosa* (diameter: 5.2 ± 0.6 μm; volume: 73.7 ± 23.4 μm^3^) and microcystin-free *M. aeruginosa* (diameter: 5.2 ± 0.5 μm; volume: 75.1 ± 22.3 μm^3^). Rotifers preferred to graze on small-sized *C. pyrenoidosa*.

**Figure 3:**
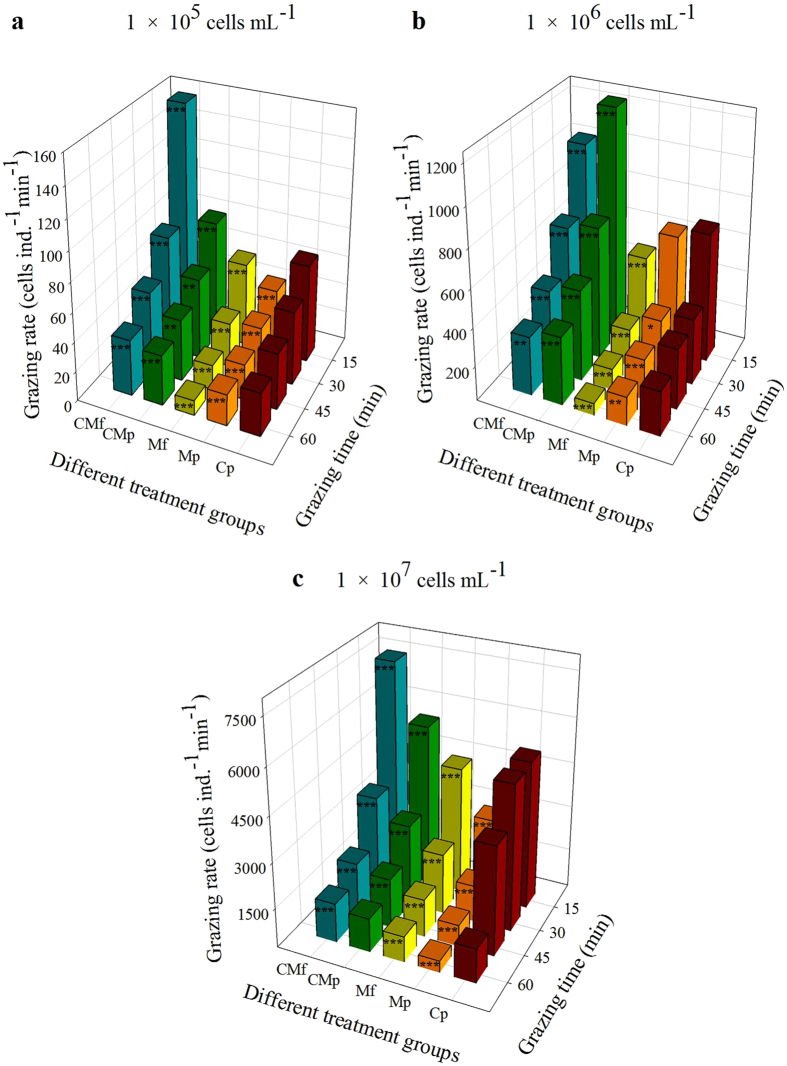
Grazing rate of rotifers over 1 h in the different treatment groups. (**a**) Changes in rotifer grazing rate at the food concentration of 1 × 10^5^ cells mL^−1^; (**b**) Changes in rotifer grazing rate at the food concentration of 1 × 10^6^ cells mL^−1^; (**c**) Changes in rotifer grazing rate at the food concentration of 1 × 10^7^ cells mL^−1^. Data were analyzed using a one-way ANOVA with the LSD test (*P < 0.05, **P < 0.01, ***P < 0.0001). All treatments were compared with *Chlorella*. The significance designations mean that there were differences between food types at a certain grazing time for a given food density.

As shown in Fig. 3, there were statistically significant differences in G at the concentrations of 1 × 10^5^, 1 × 10^6^, and 1 × 10^7^ cells mL^−1^ (one-way ANOVA, *P* < 0.01). G in mixed-diet groups increased by as much as 10–134% at the concentrations of 1 × 10^5^ cells mL^−1^ and 1 × 10^6^ cells mL^−1^ within 60 min compared to the C_p_ group (Fig. 3a,b; *P* < 0.01). The toxic and nontoxic strains of cyanobacteria inhibited the grazing intensity of rotifers at any concentration. Greater reductions of G were observed in the M_p_-group and M_f_-group (7–76%, Fig. 3a–c). The minimum G values for microcystin-producing (13 ± 2 cells ind.^−1^ min^−1^) and microcystin-free (14 ± 1 cells ind.^−1^ min^−1^) *M. aeruginosa* occurred at the concentration of 1 × 10^5^ cells mL^−1^ and at a grazing time of 60 min (Table 3). The addition of *C. pyrenoidosa* prompted an increase in rotifer grazing intensity. The G of rotifers fed a mixed diet was higher (30–75%) than those fed single cyanobacteria. The highest G value (7,522 ± 7 cells ind.^−1^ min^−1^) occurred in the CM_f_-group at the concentration of 1 × 10^7^ cells mL^−1^. In every treatment group, the highest ingestion intensity occurred at the grazing time of 15 min, whereas the lowest G occurred at the grazing time of 60 min. It decreased by 53–82% from 15 to 60 min. The G value of rotifers decreased significantly with increasing grazing time (*P* < 0.01).

## Discussion

### Effects of *M. aeruginosa* on rotifer growth

Using the model species *B. calyciflorus*, the combined effects of food concentration and food type on its life-table parameters were investigated. Microcystin-producing (PCC7806) and microcystin-free (FACHB927) *M. aeruginosa* had negative effects on the population growth of *B. calyciflorus*. The number of rotifers surviving that were cultured with microcystin-free *M. aeruginosa* was more than that cultured with microcystin-producing *M. aeruginosa*. This rotifer species was able to utilize *M. aeruginosa* at least as a supplementary source of nutrition. Similarly, *M. aeruginosa* PCC7820 was observed to decrease both the survivorship and reproduction of two freshwater rotifers *B. calyciflorus* and *Brachionus rubens*^14^. Zhao *et al*. found that *B. calyciflorus* was more sensitive to toxic *M. aeruginosa* FACHB905 than to nontoxic *M. aeruginosa* FACHB469^15^. Thus, the effects of *M. aeruginosa* on rotifer population growth depend on the strain of this cyanobacterium.

Laboratory toxicity tests involving rotifers are typically conducted with much higher food levels than occurs in the natural environment^21^. To ensure that the food density was constant when the food type varied, *Chlorella* was used as a control, while the effects of *Microcystis* on rotifers were analyzed at concentrations of 1 × 10^5^, 1 × 10^6^, and 1 × 10^7^ cells mL^−1^. Soares *et al*. studied the effects of a high concentration of *M. aeruginosa* (1 × 10^8^ cells mL^−1^) on *B. calyciflorus* growth, and confirmed that rotifers could not be killed in the short term, even in controls^22^. Although *B. calyciflorus* was tolerant of *M. aeruginosa* toxins, its survival and reproduction were adversely affected by *M. aeruginosa* PCC7806 at any concentration. The maximum concentration of toxic cyanobacteria was more harmful to rotifers than the maximum concentration of the nontoxic strain (1 × 10^7^ cells mL^−1^). *B. calyciflorus* was unable to maintain its population growth when fed high concentrations of *M. aeruginosa* alone. There were almost no offspring produced at the highest concentration of microcystin-producing *M. aeruginosa* (1 × 10^7^ cells mL^−1^). The death rate among the rotifer population was due to stress from *Microcystis* according to the experimental design. The main reasons for the decline in R_0_, r, and λ values were that the microcystin-producing strain contained toxins and the microcystin-free strain lacked nutrients. Microcystin-producing *M. aeruginosa* produces different kinds of microcystins, including hepatotoxins, neurotoxins, cytotoxins, dermatotoxins, and irritant toxins^23^. These toxins are harmful to a wide range of organisms^24^. Thus, it is possible that the toxic strain of *M. aeruginosa* contains microcystins that accumulate in rotifers after they have grazed on *Microcystis. M. aeruginosa* PCC7806 was shown to produce microcystin-LR^25^. We determined the microcystin content in *Microcystis* cells and concluded that the cause of the decrease in the rotifer population was associated with microcystins. The concentration of microcystin-LR in *M. aeruginosa* PCC7806 cells has been measured at 3.6 pg cell^−1^ using high performance liquid chromatography (HPLC)^26^. The concentration of microcystins in *B. calyciflorus* was calculated according to the G value of rotifers fed toxic cyanobacteria. The maximum content of microcystin-LR within the abdominal cavity of *B. calyciflorus* was 0.1 μg ind.^−1^, which occurred in the M_p_-group at the concentration of 1 × 10^7^ cells mL^−1^ and at a grazing time of 15 min. With the increasing concentration of microcystin-producing *M. aeruginosa*, the toxicity effect on the rotifers became more serious. The increased inhibition at higher concentrations of *M. aeruginosa* is consistent with that reported in previous studies. For example, a high concentration of *M. aeruginosa* (1 × 10^6^ cells mL^−1^) was found to affect the T, R_0_, and r of rotifers at different temperatures^27^. Complete mortality of *B. calyciflorus* cultured with toxic *M. aeruginosa* was observed at food concentrations of 50, 100, and 400 μg C L^−1^ and nontoxic *M. aeruginosa* at 400 μg C L^−1^ by Zhao^15^. Meanwhile, unknown toxic compounds of microcystin-producing *M. aeruginosa* were induced by ‘info-chemicals’ released by predators^28^. These toxins acted by inhibiting specific protein phosphatases, with which both free and covalent interactions occur, decreasing the survival, growth, and reproduction of zooplankton^29^,^30^. Toxic and nontoxic strains of cyanobacteria in the current study grew mainly as single or paired cells, which prevented the occurrence of any negative effects due to physical interference^4^. In addition to mechanical interference, the microcystin-free strain of *M. aeruginosa* is reported to be deficient in omega-3 fatty acids or potentially other lipids, which are important in zooplankton nutrition and population growth^31^. Thus, the nutrition deficiency of microcystin-free *M. aeruginosa* contributes to the poor T, R_0_, r and λ at different concentrations of food (1 × 10^5^, 1 × 10^6^, and 1 × 10^7^ cell mL^−1^), as well as the toxins produced by cyanobacteria^32^.

### Effects of *M. aeruginosa* on rotifer life history

The fundamental purpose of food consumption is to provide assimilable energy and nutrients for the maintenance, growth, and reproduction of an organism. In theory, the opportunity for rotifers to graze food particles will increase with increasing food density. If the density of algae is high enough, the rotifers should have enough food and nutrition to support their growth and movement. The results of the current study showed that the size of the rotifer population did not increase with food density in most experimental groups. The swimming speed of *B. calyciflorus* decreased in the different groups at the higher concentrations of algae (1 × 10^6^ and 1 × 10^7^ cells mL^−1^), demonstrating that the movement of rotifers has little to do with the degree of starvation^33^. Although there were statistically significant effects for some of the response variables, the magnitude of the effects on phenotypic traits was 10–30% at most, especially for variables such as body length and swimming speed. Nevertheless, the reproductive variables (time to first brood, reproductive time, post-reproductive time, and total offspring per female), which have direct effects on population growth, changed by as much as 50% compared with the control group, and interaction of food type and concentration altered these, by 50–90% in some cases. These data illustrate the obvious and dramatic influences of food concentration and type on zooplankton, both individually and in combination. Therefore, aquatic environments with a high concentration of *M. aeruginosa* will not be suitable for the growth and reproduction of *B. calyciflorus*. Given predictions that cyanobacterial blooms will increase in frequency and magnitude in the future, we anticipate that these individual responses, which will influence rotifer exponential population growth and competitive advantages, could have pronounced effects on wider populations and communities.

The reproductive time of rotifers cultured with *C. pyrenoidosa* was longer than for those fed 100% *M. aeruginosa*, indicating the inferior food quality of *M. aeruginosa* compared with the green alga *C. pyrenoidosa*. It is likely that the proportion of nutritious *C. pyrenoidosa* in the diet decreased with an increase in the relative abundance of *Microcystis*. In other words, *M. aeruginosa* ingested by rotifers did not compensate for the decrease of *C. pyrenoidosa* in terms of food availability^14^. The *M. aeruginosa* strain and concentration significantly influenced the time to first brood of rotifers, which is inconsistent with other studies, suggesting that the rotifer strain used in the current study was more sensitive compared with other strains that have been studied^14^,^19^. In terms of lifespan, rotifers that mature early result in a reduced breeding duration, affecting the total offspring produced per female. In summary, *M. aeruginosa* had toxic effects on the life-history traits of rotifers, resulting from the release of toxins from *M. aeruginosa* cells after they had been ingested by the rotifers.

### Cyanobacteria–rotifer interactions

Current models that consider collisions between predator and prey contain five major variables: the size of the perceptual field of the predator, the speed of movement of the predator, the size of the prey, the density of the prey, and the rate of movement of the prey^34^. The feeding behaviors of rotifers include two classes of activity: the rate of successful searches and the handling process. There is a suggestion in the literature that feeding behavior with regard to very small particles differs from that for larger particles^35^. The rotifer–algae interaction is related not only to food particle size, but also to food concentration. The relation between grazing intensity and food density was classified into three basic modes (linear, curve, and S type), which represent different grazing strategies of filter feeders, and showed that the diameter of food particles determines the rotifer grazing model^36^. The correlation between food concentration and grazing rate (G) was the most similar to the linear model when rotifers were fed small food particles. G decreased in a straight line as the food concentration decreased, which was in accordance with results described elsewhere^36^.

In the field, cyanobacteria occur as large, amorphous colonies (*Microcystis*) or as long, aggregated, mucilage-coated filaments (*Anabaena*) and are too large for *B. calyciflorus* to ingest, but they can be eaten by cladocerans, copepods, and herbivorous fish. These kinds of zooplankton are more sensitive to toxic cyanobacteria than rotifers. Cyanobacteria affect cladocerans more severely than they do rotifers^37^. Large cladocerans, copepods, and herbivorous fish are superior competitors in aquatic ecosystems. They are capable of suppressing or causing the extinction of rotifers through both exploitative and interference competition. The presence of cyanobacteria in aquatic plankton communities markedly affected zooplankton species structure by differentially inhibiting large zooplankton, thereby improving the survival of small rotifers^38^. *B. calyciflorus* showed a greater tolerance to *C. pyrenoidosa* than to *M. aeruginosa.* The G of rotifers fed green algae was higher than for those fed unicellular cyanobacteria. This phenomenon was observed in both single- and mixed-diet groups. Rotifers have a certain selectivity to *C. pyrenoidosa*, which leads to the conclusion that *Brachionus* feed at high rates on small spherical algae and their ingestion efficiencies are largely particle-size dependent^39^. *Microcystis* grows well in eutrophic water, partly because zooplankton prefer green algae. In addition, toxins are released into the surrounding environment when microcystin-producing *M. aeruginosa* cells degrade, which inhibits the growth of zooplankton^40^. In natural aquatic systems, *Microcystis* usually occur in large, amorphous colonies, which are too large for *B. calyciflorus* to ingest. Therefore, the G of rotifers varies according to the nutritional value of the algae, the size of the nutritive particles, and their concentration.

## Materials and Methods

### Test organisms

Two strains of *B. calyciflorus* were tested in the preliminary experiment. One strain was obtained from the Georgia Institute of Technology, GA, USA, while the other was obtained from the Nanjing Normal University, Jiangsu, China. Although the trends in the changes of the response variables were similar for both strains of *B. calyciflorus*, the strain from the USA was more sensitive to *Microcystis* than the rotifers from China. Snell reported that *B. calyciflorus* was sensitive to toxic compounds and could be used in various types of toxicity assessments^41^. To maintain the same experimental rotifer strain, we investigated the effects of toxic and nontoxic *Microcystis* on the life history characteristics of rotifers, which were provided by Dr. Snell (Georgia Institute of Technology, GA, USA) and maintained in our laboratory for more than one year before this study was initiated.

Standardization of test procedures is important in ecotoxicology, so that data collected in different labs and at different times can be compared^41^. Cysts allow test animals to be stored dried and the neonate rotifers can hatch in a physiologically uniform condition^41^. The acute toxicity test with *B. calyciflorus* using cysts to obtain test animals was accepted as a standard test procedure by the American Society for Testing and Materials (ASTM)^42^. The use of rotifers hatched from cysts makes rotifer tests among the most reliable of any aquatic invertebrate, because problems associated with the culturing of test animals are eliminated^41^.

Neonates hatched from cysts were selected as test animals to ensure the general consistency of the genotype under study. Rotifers were cultured in 1000-mL beakers containing modified Environmental Protection Agency (EPA) medium (pH = 7.8, consisting of 96 mg of NaHCO_3_, 60 mg of CaSO_4_·H_2_O, 123 mg of MgSO_4_, and 4 mg of KCl in 1 L deionized water at 25 °C)^42^. The microcystin-producing *M. aeruginosa* (PCC7806 , http://algae.ihb.ac.cn/english/Cultrues.aspx?) (M_p_), microcystin-free *M. aeruginosa* (FACHB927, http://algae.ihb.ac.cn/english/Cultrues.aspx?) (M_f_), and *C. pyrenoidosa* (C_p_) were obtained from the Institute of Hydrobiology at the Chinese Academy of Sciences. The algae were grown in 250-mL glass flasks containing a BG-11 medium formulated based on a previous study^43^. Algae showing exponential growth were harvested by centrifugation at 6000 × *g* for 15 min at 4 °C and resuspended in EPA medium. Cell densities for *C. pyrenoidosa* and *M. aeruginosa* suspensions were calculated using a hemocytometer, and then diluted to the desired concentrations with EPA medium. The rotifers and algae were incubated at 25 ± 1 °C under a 2500 lx light intensity and with a 12 L:12 D photoperiod.

### Experimental design

Rotifers were fed with *Chlorella* at a concentration of 1 × 10^6^ cells mL^−1^ prior to this study. Life strategy and grazing intensity of rotifers were evaluated under the same proportional volume of *Chlorella* and *Microcystis* for given food densities. Experiments were conducted at the following food densities: 1 × 10^5^ (total carbon content: 2.0 ± 0.1 μg C mL^−1^), 1 × 10^6^ (total carbon content: 20.6 ± 0.2 μg C mL^−1^), and 1 × 10^7^ cells mL^−1^ (total carbon content: 206.6 ± 2.9 μg C mL^−1^). Five food combinations were used: (1) C_p_-group: 100% *C. pyrenoidosa*; (2) M_p_-group: 100% microcystin-producing *M. aeruginosa* (PCC7806); (3) M_f_-group: 100% microcystin-free *M. aeruginosa* (FACHB927); (4) CM_p_-group: 50% *C. pyrenoidosa* +50% microcystin-producing *M. aeruginosa*; and (5) CM_f_-group: 50% *C. pyrenoidosa* +50% microcystin-free *M. aeruginosa*. The combinations were set to the same volume in all cases.

We used the carbon equivalent concentrations of *Chlorella* and *Microcystis*. The carbon content in phytoplankton was measured in the laboratory by the elemental analysis of carbon^44^. The carbon content of *Chlorella* per cell (5.4 ± 0.8 pg C cell^−1^) is less than that of microcystin-producing *Microcystis* (13.6 ± 4.1 pg C cell^−1^) and microcystin-free *Microcystis* (13.9 ± 3.9 pg C cell^−1^). We standardized the carbon content across food types. A linear regression for visible light absorption versus the carbon content of experimental algal species was pre-established. Based on the linear regression, algal suspensions were obtained by diluting a stock culture, which measured the light absorption at 680 nm in a spectrophotometer (Ultraspec 2100, Biochrom Ltd., Cambridge, UK) with double distilled water. The method maintained a constant total carbon nutrition among the treatments for a given food concentration.

A population-based experiment was conducted in six-microwell plates and 10 neonates (<2 h old) were placed into each well, which contained 10 mL EPA medium. The animals received the same volume of algae at 12 h intervals, with medium changes every 24 h. The number of original individuals still alive, as well as the number of neonates produced, were recorded every 12 h. The grazing intensity of the rotifers on different algae was evaluated according to grazing rate: 100 rotifers were placed in 50-mL beakers containing EPA medium. Ingested food passes through the alimentary canal of *B. calyciflorus* in about 20 min^41^. We determined the densities of rotifers and algae every 15 min for 1 h to allow a feeding interval that was than represented the gut passage time. Three replicates were performed for all treatments (*N* = 3).

An individual-based experiment was conducted in 24-microwell plates and was initiated by introducing one neonate (<2 h old) into each well, which contained 1 mL EPA medium to evaluate rotifer life-history traits. The small size of rotifers allows them to be cultured in μl volumes, and therefore toxicity can be assessed with very small amounts of test solution^41^. Any neonates later proving to be mictic instead of amictic females were discarded. Each treatment was replicated (*N* = 20). Experimental neonates were observed every 6 h under a microscope and the number of original individuals alive and the number of neonates produced were recorded. Given that *C. pyrenoidosa* is a palatable food for *B. calyciflorus* in natural freshwater ecosystems, the C_p_-group was used as the control for each experiment.

### Evaluation methods

The population growth of rotifers was evaluated using the life-table method. Generation time (T), net reproduction rate (R_0_), intrinsic growth rate (r), and finite rate of increase (λ) are the ecologically important measurements of population growth potential^41^. These variables related to survival and reproduction were calculated using equations 1,2,3,4,^45^. The intrinsic growth rate was calculated using Euler’s formula^45^:


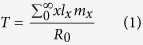



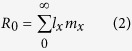



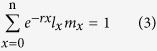



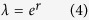


where x is the rotifer age; l_x_ is the proportion of individuals surviving at age x compared with its original cohort; and m_x_ is the mean number of female offspring produced in a unit of time by a female at age x.

The grazing rate (G) of *B. calyciflorus* was measured by the subtraction method, which quantified the microalgae concentration at the beginning and end of a feeding interval. It was calculated using equation 5^46^:


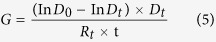


where D_0_ is the algal density in the rotifer culture solution at the beginning of the experiment (cells mL^−1^); D_t_ is the algal density in the rotifer culture solution at experimental time t (cells mL^−1^); R_t_ is the rotifer density at experimental time t (ind. mL^−1^); and t is the experimental duration (h).

In individual experiments, body length, swimming speed, time to first brood, reproductive time, post-reproductive time, and total offspring per mature rotifer female were examined to evaluate the individual life-history traits. We randomly measured the rotifer body size and swimming speed during the test period. Twenty individuals per combination of food type and food concentration were measured under a microscope (Olympus IX 51, Olympus Optical Co., Ltd., Tokyo, Japan) using an ocular micrometer (magnification x40). Videos showing the movement of rotifers and photographs were produced using cellsens standard 1.9 software (Olympus Optical Co., Ltd., Tokyo, Japan). Each short video sequence was then examined to locate the frame depicting the most outstretched individual. The body size of *B. calyciflorus* was considered to be the length from its head to toe. Rotifer motion was recorded and the movement trajectory was drawn. The video motion tracking system could perform this task automatically. The swimming speed of *B. calyciflorus* was calculated according to the slope of the movement trajectory^43^.

### Statistical analysis

In the population and individual experiments, each group sample was drawn from a normally distributed population. All samples were drawn independently of each other. Within each sample, the observations (*N* = 20) were sampled randomly and independently of each other. Factor effects were additive. Data distributions and the homogeneity of variance were tested using Kolmogorov-Smirnov and Levene’s tests, respectively. The data conformed to a normal distribution and were suitable for statistical analysis by an ANOVA. The interaction effects of food density and food type were assessed by a two-way ANOVA followed by a Tukey post-hoc test. Single food factor treatment effects were assessed by a one-way ANOVA followed by a post-hoc least significant difference (LSD) test. Probability values of *P* < 0.05 were considered statistically significant. Data were presented as means ± 1 SE, with different signals to indicate significant differences. All analyses and graphs were performed using Sigmaplot 12.5 and SPSS 22.0 for Windows.

## Conclusions

The life-strategy and grazing intensity responses of rotifers were evaluated after they had fed on different concentrations of *M. aeruginosa* mixtures. From both the population and individual experiments, we concluded that *M. aeruginosa* in combination with the green algae *C. pyrenoidosa* negatively affects the growth and reproduction of *B. calyciflorus*. Rotifers are able to utilize *M. aeruginosa* as a food source despite its inadequate nutrition, but unicellular *M. aeruginosa* cultured in the laboratory is a poor food for aquatic herbivores and contains toxic substances that negatively affect rotifers. The results of the grazing intensity experiment suggested that *B. calyciflorus* prefers to graze on *C. pyrenoidosa*. Food intake by *B. calyciflorus* is associated with the particle size and species of algae. These findings not only help us to understand the response of rotifers to environmental toxins, but also contribute to the development of aquacultural systems for these organisms.

## Additional Information

**How to cite this article:** Liang, Y. *et al*. Life strategy and grazing intensity responses of *Brachionus calyciflorus* fed on different concentrations of microcystin-producing and microcystin-free *Microcystis aeruginosa. Sci. Rep.* **7**, 43127; doi: 10.1038/srep43127 (2017).

**Publisher's note:** Springer Nature remains neutral with regard to jurisdictional claims in published maps and institutional affiliations.

## Figures and Tables

**Table 1:** Survivorship data for rotifers in the life-table experiment.

Food concentration (cells mL^−1^)	Time (h)	S (ind.) (C_p_-group)	S (ind.) (M_p_-group)	S (ind.) (M_f_-group)	S (ind.) (CM_P_-group)	S (ind.) (CM_f_-group)
1 × 10^5^	24	7 ± 3	8 ± 1	9 ± 1	8 ± 2	9 ± 1
	48	6 ± 3	5 ± 2	6 ± 1	5 ± 1	7 ± 2
	72	5 ± 2	4 ± 1	3 ± 2	3 ± 2	6 ± 2
	96	3 ± 2	0	2 ± 1	0	2 ± 1
	120	1 ± 1	0	0	0	0
1 × 10^6^	24	7 ± 2	6 ± 1	8 ± 1	4 ± 1	4 ± 1
	48	7 ± 1	5 ± 1	6 ± 2	3 ± 1	3 ± 1
	72	7 ± 2	3 ± 1	5 ± 2	2 ± 1	2 ± 1
	96	6 ± 1	2 ± 1	3 ± 1	0	2 ± 1
	120	4 ± 1	0	0	0	1 ± 1
	144	3 ± 1	0	0	0	0
1 × 10^7^	24	8 ± 2	6 ± 1	8 ± 1	6 ± 1	4 ± 1
	48	6 ± 3	3 ± 1	6 ± 2	5 ± 1	3 ± 1
	72	6 ± 2	2 ± 1	4 ± 1	2 ± 1	2 ± 1
	96	3 ± 1	0	3 ± 1	0	1 ± 1
	120	2 ± 1	0	0	0	0

Data are means ± SD (n = 3). S: the number of surviving rotifers; C_p_-group: *Chlorella pyrenoidosa*; M_p_-group: microcystin-producing *Microcystis aeruginosa* (PCC7806); M_f_-group: microcystin-free *M. aeruginosa* (FACHB927); CM_p_-group: the mixture of *C. pyrenoidosa* and microcystin-producing *M. aeruginosa*; CM_f_-group: the mixture of *C. pyrenoidosa* and microcystin-free *M. aeruginosa*.

**Table 2:** Grazing rate of *B. calyciflorus* in single-diet groups.

Food concentration (cells mL^−1^)	Time (min)	G (cells ind.^−1^ min^−1^) (C_p_-group)	G (cells ind.^−1^ min^−1^) (M_p_-group)	G (cells ind.^−1^ min^−1^) (M_f_-group)
1 × 10^5^	15	67 ± 1	44 ± 2	57 ± 1
	30	50 ± 1	33 ± 2	30 ± 1
	45	38 ± 1	24 ± 1	17 ± 1
	60	29 ± 1	21 ± 1	10 ± 1
1 × 10^6^	15	701 ± 14	649 ± 25	495 ± 36
	30	366 ± 13	314 ± 32	218 ± 14
	45	336 ± 10	231 ± 46	130 ± 11
	60	251 ± 6	169 ± 33	82 ± 15
1 × 10^7^	15	5135 ± 1	2930 ± 1	4419 ± 15
	30	5064 ± 31	1408 ± 7	2196 ± 8
	45	3809 ± 5	902 ± 2	1441 ± 12
	60	1322 ± 12	580 ± 5	1043 ± 5

Data are means ± SD (n = 3). G: grazing rate of rotifers; C_p_-group: *C. pyrenoidosa*; M_p_-group: microcystin-producing *M. aeruginosa* (PCC7806); M_f_-group: microcystin-free *M. aeruginosa* (FACHB927).

**Table 3:** Grazing rate of *B. calyciflorus* for *C. pyrenoidosa* and *M. aeruginosa* in mixed-diet groups.

Food concentration (cells mL^−1^)	Time (min)	G (CM_p_-group) (cells ind.^−1^ min^−1^)		G (CM_f_-group) (cells ind.^−1^ min^−1^)	
		G (C_p_)	G (M_p_)	G (C_p_)	G (M_f_)
1 × 10^5^	15	54 ± 2	26 ± 3	109 ± 1	48 ± 3
	30	37 ± 2	17 ± 2	53 ± 1	24 ± 1
	45	28 ± 1	14 ± 1	37 ± 2	18 ± 1
	60	18 ± 1	13 ± 2	22 ± 2	14 ± 1
1 × 10^6^	15	677 ± 32	563 ± 31	596 ± 35	424 ± 41
	30	386 ± 56	326 ± 43	423 ± 42	255 ± 8
	45	264 ± 19	191 ± 36	275 ± 21	137 ± 20
	60	188 ± 12	142 ± 20	203 ± 15	60 ± 16
1 × 10^7^	15	4472 ± 4	702 ± 3	4058 ± 2	2654 ± 9
	30	2198 + 4	627 ± 23	2149 ± 2	1047 ± 15
	45	1280 ± 26	430 ± 7	1157 ± 12	896 ± 19
	60	798 ± 22	305 ± 4	767 ± 13	703 ± 3

Data are means ± SD (n = 3). G: grazing rate of rotifers; C_p_: *C. pyrenoidosa*; M_p_: microcystin-producing *M. aeruginosa* (PCC7806); M_f_: microcystin-free *M. aeruginosa* (FACHB927); CM_p_-group: the mixture of *C. pyrenoidosa* and microcystin-producing *M. aeruginosa*; CM_f_-group: the mixture of *C. pyrenoidosa* and microcystin-free *M. aeruginosa*.
